# Correspondence

**Published:** 1910-03

**Authors:** T. D. Crothers

**Affiliations:** Editor and Secretary; Hartford, Conn.


### Correspondence.

Established in 1876.
QUARTERLY JOURNAL OF INEBRIETY.
The Organ of the American Association for the Study of Inebriety and Alcohol.
T. D. CROTHERS, M. D., Editor and Secretary.

HARTFORD, CONN., February 10, 1910.

MY DEAR DR. DANIEL: I want to assure you of my keen appreciation of your exceedingly kind note in the February number of our work and Senate Document No. 48.

Many editors have failed to appreciate the significance of the public recognition (by Congress) of the scientific side of the drink problem, and have regarded this document as a mere accident, whereas in reality it is the first shot of a great revolution that will extend through the entire profession with the result of bringing the subject into the realm of science, and study, and treating it from the basis of facts and not theories.

The intense conservatism of the profession is sad, indeed, and as a result a great neurotic disease and psychosis that is curable to a very high degree is left entirely to laymen and quacks.

They have recognized the reality of the physical and mental side. The quacks by exploiting certain remedies with pretention and mystery, the laymen by mental and moral remedies, and both recognize the need of education and recognition of the everyday facts of life.

The trained physician who is, of all persons, most competent, is not seen as a teacher or leader, except in individual instances. It is a great pleasure to recognize that you and your work have caught sight of the facts and have urged the dawn of the new day that is coming, and I thank you again for your most cheery words, and far-reaching discernment of the great on-coming tide of a new range of practice, a new field for the benefit of humanity.

Command me any time and believe me,

Very sincerely yours,

T. D. Crothers.

[This refers to the publication by the U. S. Senate, and promulgation at government expense, of Senate Document 48, being the papers read and conclusions reached at the March meeting of the National Association for the Study of Alcohol. A copy can be had free by sending a postal card request to any Senator.—Daniel.]

